# Diabetes care in Switzerland: good, but perfectible: a population-based cross-sectional survey

**DOI:** 10.1186/1472-6963-13-232

**Published:** 2013-06-25

**Authors:** Isabelle Peytremann-Bridevaux, Julie Bordet, Bernard Burnand

**Affiliations:** 1 Institute of Social and Preventive Medicine (IUMSP), Lausanne University Hospital, Lausanne, Switzerland

**Keywords:** Healthcare quality, Diabetes, Cross-sectional study

## Abstract

**Background:**

While Switzerland invests a lot of money in its healthcare system, little is known about the quality of care delivered. The objective of this study was to assess the quality of care provided to patients with diabetes in the Canton of Vaud, Switzerland.

**Methods:**

Cross-sectional study of 406 non-institutionalized adults with type 1 or 2 diabetes. Patients’ characteristics, diabetes and process of care indicators were collected using a self-administered questionnaire. Process indicators (past 12 months) included HbA1C check among HbA1C-aware patients, eye assessment by ophtalmologist, microalbuminuria check, feet examination, lipid test, blood pressure and weight measurement, influenza immunization, physical activity recommendations, and dietary recommendations. Item-by-item (each process of care indicator: percentage of patients having received it), composite (mean percentage of recommended care: sum of received processes of care / sum of possible recommended care), and all-or-none (percentage of patients receiving all specified recommended care) measures were computed.

**Results:**

Mean age was 64.4 years; 59% were men. Type 1 and type 2 diabetes were reported by 18.2% and 68.5% of patients, respectively, but diabetes type remained undetermined for almost 20% of patients. Patients were treated with oral anti-diabetic drugs (50%), insulin (23%) or both (27%). Of 219 HbA1C-aware patients, 98% reported ≥ one HbA1C check during the last year. Also, ≥94% reported ≥ one blood pressure measurement, ≥ one weight measurement or lipid test, and 68%, 64% and 56% had feet examination, microalbuminuria check and eye assessment, respectively. Influenza immunization was reported by 62% of the patients.

The percentage of patients receiving all processes of care ranged between 14.2%-16.9%, and 46.6%-50.7%, when considering ten and four indicators, respectively. Ambulatory care utilization showed little use of multidisciplinary care, and low levels of participation in diabetes-education classes.

**Conclusions:**

While routine processes-of-care were performed annually in most patients, diabetes-specific risk screenings, influenza immunization, physical activity and dietary recommendations were less often reported; this was also the case for multidisciplinary care and participation in education classes. There is room for diabetes care improvement in Switzerland. These results should help define priorities and further develop country-specific chronic disease management initiatives for diabetes.

## Background

Managing chronic diseases, which account for a large mortality, morbidity and disability burden within communities, requires better integration and coordination of care. Targeting this goal, the Chronic Care Model promotes teamwork, self-management education for patients, evidence-based care, and organization of patients’ and professionals’ exchange and transmission of information as several important components [1,2]. The transfer and implementation of evidence-based knowledge in practice is nevertheless challenging, and the quality of care for chronic diseases remains sub-optimal [3-6]. Diabetes care is not an exception. Indeed, several population- [7-12] and physician practice-based studies [13-15] from North American and European countries have shown that there is much room for improvement in both processes and outcomes of diabetes care. Some countries have nevertheless already noticed quality of care improvements [16-18].

Switzerland is a federal and democratic state comprising of 26 cantons, with about 8 millions residents. The 1996 Law on Health Insurance stipulated that all Swiss residents must purchase basic health insurance, which covers a comprehensive basket of goods and services. Ambulatory care is provided by primary care physicians and specialists working mostly independently in solo and small group private practices. Except for insurees opting for a health insurance plan limiting access to specialists (gate-keeping principle), in return of lower premiums, Swiss residents have unlimited access to ambulatory care. Hospitals, which provide inpatient care, can also provide general and specialist ambulatory care. Switzerland spends around 11% of its GDP on health [19]. The returns on this investment are an average life-expectancy above European average, low mortality rates, and residents’ overall satisfaction with the Swiss health care system [20,21]. However, data collection, particularly in the ambulatory care sector, remains insufficient to address whether “Switzerland receives value for money for its major financial investment in healthcare” [21].

Data on the quality of diabetes care is scarce in Switzerland. In 1995, a survey showed that several diabetes-specific checks were self-reported by only a small fraction of patients with diabetes (microalbuminuria check, glycemic self-controls, HbA1C, feet examination, respectively 13%, 39%, 53% and 54%) [22]. In 2004, improvements were shown among patients from a convenience sample of primary care physicians [23], and the first population-based measure of prevalence of diabetes was found to be around 7% [24]. However, quality of care data are still infrequently and unsystematically collected. In addition, when they are available, they focus on intermediate outcomes, as well as clinical and biological cardiovascular risk factors [25-27]. Within the development of a regional diabetes program (“Programme cantonal Diabète”) [28], we conducted a survey to assess the characteristics of patients with diabetes as well as the quality of the care they receive. Unlike previous studies on the quality of care in Switzerland, this study was population-based, considered a larger number of indicators and combined both patients’ and physicians’ reported data. The aim of the current paper is to present results of process of care indicators, as reported by patients, individually and in combination.

## Methods

### Study design

This cross-sectional study, conducted in the fall of 2011, used self-administered paper questionnaires for data collection.

### Setting, participants and recruitment

Patients with diabetes were recruited by community-based pharmacies registered in the canton of Vaud, Switzerland, a large French-speaking canton with over 720’000 inhabitants. Sample size calculations were performed to estimate the number of patients with diabetes we would need to recruit to obtain good precision (i.e. confidence interval width) of process of care indicators (% of patients with specific annual checks); to be conservative, sample size was calculated around a 50% point estimate. Taking into account the clustering of data by pharmacies (40 pharmacies, each recruiting 15 patients, intra-class correlation 0.05, alpha 0.05, beta 0.2), 600 diabetic patients were considered sufficient. Therefore, we decided to contact 140 pharmacies to get a minimum of 40 willing to participate. They were randomly selected from a total of 241 pharmacies registered in the canton in April 2011; 56 finally agreed to participate (participation rate of 40%) [29]. Patients were eligible if they came to the pharmacy with a prescription for oral anti-diabetic medications (OAD), insulin, glycemic strips or glucose meter, were aged ≥18 years and non-institutionalized, and known to have a diagnosis of diabetes for at least 12 months. Patients not residing in the canton of Vaud, not speaking or understanding French well enough, or those presenting with an obvious cognitive impairment were excluded, as well as women with gestational diabetes. Out of 1013 eligible patients, 809 accepted to receive the questionnaire and 408 filled it out and returned it; two patients were excluded because their treating physicians reported a major cognitive impairment. The final analytical sample therefore consisted of 406 patients, corresponding to a participation rate of 50.2% (406/809). Since we collected both patients’ and physicians’ reported data, treating physicians (n = 186) were also contacted, with patients’ consent; almost two-thirds (60%) of them agreed to fill in a brief questionnaire. Agreement between patients’ and physicians’ report of process of care measures, as measured by uniform kappa, was shown to be good for routine process of care (measurement of blood pressure, HbA1c, weight, and lipid profile), and less satisfactory for microalbuminuria check, foot examination and eye assessment (personal communication, TH Collet).

### Measure

Data of interest for the current study included self-reported data targeting patients’ characteristics and health status (age, gender, socio-economic and insurance status, place of residence, smoking status, alcohol consumption using the AUDIT-C questionnaire [30], physical activity levels using questions from the Swiss Health Survey [31], weight and height, self-rated health and comorbidities), and the description of their diabetes (type of diabetes, disease duration, treatment, diabetes-related complications). The following ten primary process of care indicators were also collected (with reference to the past 12 months): HbA1C check among those who reported knowing what HbA1C was (yes,1×/year; yes,>1×/year; no; unknown), eye assessment by ophthalmologist (yes,<1 year ago; yes,1-2 years ago; yes, >2 years ago; never; unknown), annual microalbuminuria check (yes; no; unknown), annual feet examination by physician (yes; no; unknown), annual lipid test (yes; no; unknown), blood pressure measure (yes,1×/year; yes, 2-3×/year; yes, ≥4×/year; no; unknown), annual weight measure (yes; no; unknown), annual influenza immunization (yes; no; unknown), physical activity recommendations (yes; no; unknown), written or verbal diet recommendations (yes; no; unknown). Finally, we asked about secondary process of care measures: i) ambulatory care visits in the prior 12 months (yes,1×; yes,2-3×; yes,≥ 4×; no) with the following providers, primary care physicians (general internal medicine, family medicine or general practice), diabetologists, nurse specialists, dieticians and podiatrists, and ii) participation in self-management education classes (yes, <1 year ago; yes,1-2 years ago; yes, >2 years ago; never; unknown).

We used three approaches to assess process of care performance [32]:

•item-by-item: for each single process of care indicator, percentage of patients having received it

•composite: mean percentage of recommended care as the sum of received process of care divided by the sum of possible recommended care

•all-or-none: percentage of patients receiving all specified recommended care

Combined measures of indicators have been recommended when item-by-item results are already good [32]. In this paper, we will refer to the latter two (composite and all-or-none) as combined measures of receipt of services during the past 12 months.

The combined measures of process of care indicators were the following: all ten indicators, six “diabetes-specific” indicators (HbA1C check, eye assessment by ophtalmologist, micro-albuminuria test, feet examination, lipid test, influenza immunization, excluding measures that are normally proposed to any patient such as blood pressure and weight measurement, physical activity and diet recommendations), and the four indicators considered in the 2008 International Commonwealth Fund Survey (HbA1C check, eye assessment, feet examination, lipid test) [4]. Because of a skip question survey method, which limited answers about receipt of HbA1C tests to those reporting knowing what HbA1C was (HbA1C-aware patients), we restricted the analysis of combined measures of receipt of services (that included HbA1C checks) to this HbA1C-aware sub-group. In order not to exclude HbA1C unaware patients from all analysis, we also constructed two combined measures not considering HbA1C check: all processes of care except HbA1C check (n = 9), and five “diabetes-specific” indicators (all six “diabetes-specific” indicators mentioned above, except HbA1C check).

### Statistical analysis

Descriptive analyses of primary process of care indicators with confidence intervals around point estimates (item-by-item measurement) were conducted, taking into account the hierarchical structure of the data (clusters of pharmacies). Percentages of receipt of processes of care were based on those who answered the particular question and not on the total number of participants (complete case analysis). The exact number of patients considered in the analysis is specified in all tables.

For sensitivity testing, item-by-item analyses were then performed assuming worst case and best case scenarios. In worst case scenarios, we considered patients having not responded to the question (missing data) or having chosen “don’t know”, as patients not having received the process of care during the past 12 months. In best case scenarios, we considered these same patients as having received the process of care during the past 12 months.

Analyses of combined measures were both performed for complete cases and to take into account cases with missing and “don’t know” data. Including missing and “don’t know” answers allowed us to consider all patients, and thus to estimate the lowest (minimal) percentage of patients who would have received at least a specified number of processes of care. The highest (maximal) percentage of possible patients receiving the specified number of processes of care was calculated with “don’t know” answers and missing data counted as receipt of care. These lowest (minimal) and highest (maximal) percentages therefore represent the range of values within which the percentage of patients having received these processes would lie when considering all targeted patients. STATA 11.0 was used for all analyses.

Ethical approval was received from the Cantonal Ethics Committee of Research on Human Beings of the Canton of Vaud (“Commission cantonale (VD) d’éthique de la recherche sur l’être humain”, Protocol N° 151/11). Written informed consent was obtained from all participants, and data were kept anonymous and confidential.

## Results

Descriptive information about the study population is shown in Table 1. Briefly, mean age was 64.4 years and the majority of participants (59%) were men. While 16% were current smokers, 82% were either overweight or obese, half were considered to engage in at-risk drinking behaviour (AUDIT-C scores ≥ 3 for women and ≥ 4 for men), and almost one-third reported being physically inactive. Type 1 and type 2 diabetes were reported by 18.2% and 68.5% of patients, respectively. Description of diabetes type remained undetermined for almost 20% of patients. The vast majority of patients reported being treated with anti-diabetic drugs and/or insulin, and at least one complication of diabetes was reported by nearly half of all patients. Glucose self-monitoring was performed by 82% of the patients, and 54% of participants indicated knowing what HbA1C was. Table 2 displays further detailed information on diabetes characteristics.

**Table 1:** **Characteristics of participants** (**n** = **406 diabetic patients**)

Age (n = 406), mean (SD)	64.4 (11.4)
Women (n = 406)	40.6%
Civil status (n = 403)	
Single	8.7%
Married/partnership	62.5%
Divorced/separated/widowed	28.8%
Education (n = 392)	
Primary	19.1%
Secondary	55.6%
Tertiary	25.3%
Employment status (n = 394)	
Full-time	25.1%
Part-time	9.1%
Retired	55.6%
Unemployment/handicapped/student	5.8%
Stay-at-home	4.3%
Place of residence (n = 399)	
Urban	38.9%
Semi-urban	27.1%
Rural	34.1%
Current smoking (n = 398)	16.3%
BMI (n = 378)	
Overweight	35.7%
Obese	46.3%
Physically inactive (n = 385)	28.6%
Self-reported health (n = 398)	
Excellent/very good	15.9%
Good	64.3%
Medium/poor	19.9%

**Table 2:** Diabetes characteristics

Type of diabetes (n = 406)	
Type 1	12.8%
Type 2	68.5%
Undetermined	18.7%
Duration of the disease (n = 399)	
1-5 years	28.3%
6-10 years	23.1%
11-15 years	17.3%
≥16 years	31.3%
Type of treatment (n = 405)	
Oral antidiabetic drugs (OAD)	49.6%
Insulin	22.7%
Oral antidiabetic dugs and insulin	26.9%
None/unknown	0.8%
Glucose self-monitoring (n = 398)	82%
Diabetes complications (n = 396)†	
At least one	46.7%
Macrovascular complications*	34.9%
Microvascular**	24%

†: Myocardial infarction/angina, stroke, retinopathy (excluding cataract and glaucoma problems), nephropathy (including dialysis or renal transplantation), neuropathy (lower limb pain or sensibility problems/ulcer/amputation), severe hypo or hyperglycemia.

* Myocardial infarction/angina, stroke, neuropathy (lower limb pain or sensibility problems/ulcer/amputation).

** Retinopathy, nephropathy (including dialysis or renal transplantation).

In item-by-item analysis for receiving at least one specific process of care during the past 12 months, HbA1C check was above 90% among those who reported knowing what HbA1C was. Similar high percentages were found for routine clinical tests like blood pressure and weight measurements, as well as lipid tests. Item-by-item results remained high for HbA1C check and blood pressure measurement even when raising the bar to two or more screens in the past year. Under these parameters, HbA1C and blood pressure checks were reported by 83.4% (95% CI 77%-88.3%) and 86.5% (83.1%-89.4%) of the patients, respectively. Physical activity recommendation, feet examination, microalbuminuria test and influenza vaccination (in descending order of frequency) were performed less often. While 64% of patients reported having a microalbuminuria test carried out, it is worth noting that 14.3% of the patients answered “don’t know” to that specific question. Eye assessment by an ophthalmologist and dietary recommendations were the process of care indicators that ranked the lowest, with only 56% and 49% of the patients reporting them, respectively (Table 3). However, report of eye assessment by an ophthalmologist increased to 73.5% (69.4%-77.3%) when considering a two-year timeframe. Except for those processes of care with high numbers of “don’t know” answers, worst and best case scenarios results remained within the 95% confidence interval of point estimates.

**Table 3:** **Primary process of care indicators** (**item**-**by**-**item**): **receipt of service during past 12 months**, **by decreasing order of receipt** %

	**N for each response modality**	**Receipt of service**	**(95% CI)**	Worst case scenario**	**Best case scenario†**
≥ one HbA1C check (n = 218)*	Yes: 214	**98**.**1%**	**(95.3%-98.9%)**	97.7%	99.1%
	No: 2				
	DK: 2				
≥ one blood pressure measurement (n = 399)	Yes: 388	**96**.**4%**	**(95**.**7%-97**.**0%)**	95.6%	98.0%
	No: 8				
	DK: 3				
≥ one weight measurement (n = 396)	Yes: 374	**94**.**4%**	**(91.4%-96.6%)**	92.1%	94.8%
	No: 21				
	DK: 1				
≥ one lipid test (n = 401)	Yes: 378	**94**.**2%**	**(91.6%-96.1%)**	93.1%	96.8%
	No: 13				
	DK: 10				
Physical activity recommendations, written or verbal (n = 398)	Yes: 277	**69**.**6%**	**(65.2**-**71.1%)**	68.2%	70.4%
	No: 120				
	DK: 1				
Diabetic foot examination by a physician (n = 397)	Yes: 265	**66**.**8%**	**(61.5%-72**.**9%)**	65.3%	68.2%
	No: 129				
	DK: 3				
≥ one urine test (for micro-albuminuria) (n = 399)	Yes: 252	**63**.**2%**	**(57.6%-70%)**	62.1%	77.8%
	No: 90				
	DK: 57				
Influenza immunization (n = 402)	Yes: 250	**62**.**2%**	**(58.6%-65.5%)**	61.6%	62.8%
	No: 151				
	DK: 1				
Eye assesment by ophthalmologist (n = 399)	Yes: 225	**56**.**4%**	**(51.4%-61%)**	55.4%	59.1%
	No: 166				
	DK: 8				
Diet recommendations, written or verbal (n = 399)	Yes: 194	**48**.**6%**	**(43.2%-54.2%)**	47.8%	50.0%
	No: 203				
	DK: 2				

*DK* don’t know.

* Among HbA1C aware patients (skip question).

** Don’t know answers and missing data assumed as “no receipt” of process of care.

† Don’t know answers and missing data assumed as “receipt” of process of care.

All combined results (composite and all-or-none) are shown in Table 4. Overall, while patients with diabetes received 80% of processes of care (i.e. 8 out of 10, or 5 out of 6, or 3 out of 4 processes of care), all processes of care were received by only less than 20% of patients. In addition, the mean percentage of care received varied between 72% and 85%, depending on whether HbA1C check was, or not, considered in the combination of indicators. Upon looking at combined measures which incorporated HbA1C checks (i.e. among HbA1C-aware patients), the percentage of patients receiving all processes of care ranged from 14.2% to 16.9% when all 10 processes of care were targeted, and 46.6% to 50.7% when the list was restricted to four indicators. Complete case analyses of all-or-none indicators showed similar results.

**Table 4:** **Primary process of care indicators**: **combined measures of receipt of service during past 12 months**, % (**n**)

**Combined measures among patients who reported knowing what HbA1C is (i.e. among HbA1C-aware patients, n=219)**		
1) All 10 processes of care §		
Complete cases*	Composite: mean % of recommended care (SD)	**82% (13.5)**
(n=175)	All-or-none: receipt of10/10 care processes, % (n)	**17.7% (31)**
All patients (n=219)	Lowest ** % (n) – Highest*** % (n) of patients receiving 10/10 care processes	**14.2% (31) - 16**.**9% (37**)
	Lowest ** % (n) – Highest*** % (n) of patients receiving at least 8/10 care processes	**65**.**3% (143) - 74.4% (163)**
2) Six “Diabetes-specific” processes of care †		
Complete cases *	Composite: mean % of recommended care (SD)	**82.4% (15.5)**
(n=182)	All-or-none: receipt of 6/6 care processes, % (n)	**30.2% (55)**
All patients (n=219)	Lowest ** % (n) – Highest*** % (n) of patients receiving 6/6 care processes	**25.1% (55) - 28.8% (63)**
	Lowest ** % (n) – Highest*** % (n) of patients receiving at least 5/6 care processes	**64.4% (141) - 71.2% (156)**
3) Restricted list of four processes of care ‡		
Complete cases*	Composite: mean % of recommended care (SD)	**85.3% (16.2)**
(n=204)	All-or-none: receipt of 4/4 care processes, % (n)	**50% (102)**
All patients (n=219)	Lowest ** % (n) – Highest*** % (n) of patients receiving 4/4 care processes	**46.6% (102) - 50.7% (111)**
	Lowest ** % (n) – Highest*** % (n) of patients receiving at least 3/4 care processes	**88.6% (194) - 90.9% (199)**
**Combined measures without consideration of HbA1C check (i.e. among all patients**, **irrespective of their HbA1C knowledge status, n=406)**		
1) Nine processes of care (all 10 except HbA1C) §§		
Complete cases*	Composite: mean % of recommended care (SD)	**75.3% (18)**
(n=304)	All-or-none: receipt of 9/9 care processes, % (n)	**12.5% (38)**
All patients (n=406)	Lowest ** % (n) – Highest*** % (n) of patients receiving 9/9 care processes	**9.4% (38) - 12.3% (50**)
	Lowest ** % (n) – Highest*** % (n) of patients receiving at least 7/9 care processes	**53% (215) - 65.8% (267)**
2) Five “Diabetes-specific” process of care (all six “Diabetes-specific” except HbA1C) ††		
Complete cases*	Composite: mean % of recommended care (SD)	**72.6% (22.7)**
(n=319)	All-or-none: receipt of 5/5 care processes, % (n)	**24.1% (77)**
All patients (n=406)	Lowest ** % (n) – Highest*** % (n) of patients receiving 5/5 care processes	**19% (77) - 23.4% (95)**
	Lowest ** % (n) – Highest*** % (n) receiving at least 4/5 care processes	**49.5% (201) - 59.9% (243)**

§ HbA1C check (among HbA1C-aware patients who answered the HbA1C process of care question), eye assessment, microalbuminuria check, feet examination, lipid test, blood pressure and weight measurement, influenza immunization, physical activity and diet recommendations.

* Only results for cases that are “complete”: cases with “don’t know” answers and cases with missing data are dropped from analysis.

** Lowest (minimal) percentage of patients having received at least a certain number of processes of care where “don’t know” and missing data are assumed as “not having received the process of care”.

***Highest (maximal) percentage of patients having received at least a certain number of processes of care where “don’t know” and missing data are assumed as “having received the process of care”.

† HbA1C check (among HbA1C-aware patients who answered the HbA1C process of care question), eye assessment, microalbuminuria check, feet examination, lipid test, influenza immunization.

‡ HbA1C check (among the HbA1C-aware and thus answered the HbA1C process of care question), eye assessment, feet examination, lipid test (diabetes indicator used in the 2008 Commonwealth Fund survey).

§§ Eye assessment, micro-albuminuria test, feet examination, lipid test, blood pressure measure, weight measure, influenza immunization, physical activity recommendations, diet recommendations.

†† Eye assessment, microalbuminuria check, feet examination, lipid test, influenza immunization.

Combined measures excluding HbA1C checks showed higher percentages of patients receiving all services when the number of services considered was lowered (9.4% to12.3% of patients received nine out of nine services while 19% to 23.4% of patients received five out of five services). Higher percentages of receipt of services were also found when conducting complete case analysis of all-or-none measures. Finally, restricting analyses to HbA1C-aware patients yielded slightly better results (14.2%-16.9% and 25.6%-29.2%, respectively, for nine out of nine and five out of five services, for example).

**Figure 1:**
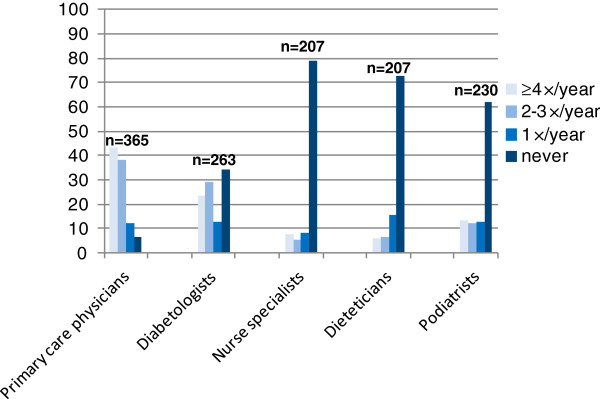
**Percentage of ambulatory care visits (number of times/year) to various healthcare professionals.**

Ambulatory care utilization data showed that, while almost all patients reported visiting a primary care physician in the previous 12 months (93.4%), one-third of them did not see a specialist (diabetologist-endocrinologist), and between 2/3 and 3/4 of respondents did not consult with other diabetes-related healthcare professionals (diabetic nurse specialists, dieticians, or podiatrists) during the past year (Figure 1). Lastly, attendance at self-management education classes (group or individual sessions) was low; only one third reported having ever participated in this type of class.

## Discussion

While routine clinical and laboratory tests (blood pressure and weight measurements, lipid tests) were performed annually in most patients with diabetes, risk screenings such as feet examination, microalbuminuria check and eye assessment, as well as physical activity and dietary recommendations, and influenza immunization, were less often reported. Also only a minority of patients reported having received all processes of care. In addition, a substantial number of patients did not know the meaning of HbA1c and quite a few did not specify the type of diabetes they live with. There was also little evidence for multidisciplinary care, low referrals to non-physician diabetes healthcare professionals, and low reporting of patient participation in diabetes education sessions.

The results of our study are similar to past findings. However, differences in healthcare organization structure and variations in measurement of process of care indicators limit the scope of such comparisons. Most published results have included self-reported data which point to better item-by-item outcomes for blood pressure, lipid and HbA1C controls than for annual eye assessment, microalbuminuria check, feet examination, and influenza vaccination [8,10,12,16,33-35]. Similar indices were found by the European Core Indicators in Diabetes (EUCID) project, which gathered representative regional or national quality of care data over 19 countries [5]. These differences could be explained by the fact that it is easier to obtain better results for processes of care that are recommended to be performed more than once a year (blood pressure controls, HbA1C and lipids checks) than for those recommended once a year or less (microalbuminuria, check and feet examination, for example).

The 2008 Commonwealth Fund population-based survey collected data on care experiences of sick patients residing in eight high income countries (Australia, Canada, France, Germany, Netherlands, New-Zealand, United Kingdom, United States) [4]. While only targeting a restricted set of diabetes process of care indicators (HbA1C check, lipid test, eye assessment and feet examination), the care of Dutch and British patients consistently ranked among the best, even when considering item-by-item and all-or-none measures. While 31% and 36% of French and Australian patients reported all four processes of care, up to 59% and 67% of Dutch and British patients received all four, respectively. Results from a similarly constructed indicator among our HbA1C-aware patients (Table 4) demonstrated that Swiss performance would probably end up in the middle, very close to German and US results.

Combined measures of indicators (such as all-or-none) are recommended when item-by-item results are already good, because they help identify areas of improvement, particularly when processes of care are considered [32]. However, a multitude of combinations can be used potentially. Also, good all-or-none results are much harder to achieve compared to results that measure outcomes using partial completion of indicators. Examples from the literature show that in the US, while less than 5% of respondents with diabetes from the 1994 Behavioral Risk Factor Surveillance System (BRFSS) met all five standards of care [12], approximately one out of four similar patients received adequate care as measured by the receipt of nine out of eleven diabetes care processes in 2009 [8]. There was a greater variation reported in the national audit of diabetes care in England with 10% of patients reporting three out of four processes of care (smoking cessation recommendations, HbA1C, blood pressure and lipid checks), 70% of the patients signaling receipt of the following three services (blood pressure, lipid and HbA1C controls) and 50% of patients reporting nine out of nine processes of care [33]. Comparisons of study results are difficult to interpret however, as combinations and measurements of indicators varied between studies. In fact, less cross-country variations were shown in the 2008 Commonwealth Fund comparison [4], which used the same methodology in all countries. Such variations of results could also be explained by the fact that a careful selection of indicators is needed when considering all-or-none measures. Indeed, Nolan et al. recommended that the number of indicators remains small (four to eight), and that they are considered as important measures and be consensually accepted as the basis of good care for a given condition. Also, all-or-none measures magnify measurement errors since “one unreliable component measure will contaminate the whole score” [32].

The main strengths of this study are that we used a population-based sample of patients with diabetes, and targeted a range of recommended diabetes process of care indicators. Of course, the first limitation to our results is the use of self-reported data. Indeed, the accuracy of self-report depends on the type of data collected, and patients with diabetes may over- or underestimate elements of the care process [36,37]. However, complementary analyses of our data suggested acceptable agreement between patients’ reported process of care measures and primary care physicians’ report. Indeed, while agreement, as measured by uniform kappa, was good for past 12 months routine process of care such as measurement of blood pressure, HbA1c, weight, and lipid profile, it was less satisfactory for procedures such as microalbuminuria check, foot examination and eye assessment [TH Collet, personal communication]. The second limitation is the use of “one check in the past 12 months” time frame for some process of care indicators, which can be questioned. However, expert groups currently accept this time frame for population-based quality assessment purposes, which facilitates population-level comparisons [5,38]. The third limitation is the somewhat smaller than expected sample size. Because we had more clusters (pharmacies) than we expected, and because of our conservative sample size calculations, the precision around point estimates was nevertheless acceptable [29]. The fourth limitation relates to the representativeness of our sample of patients with diabetes, which may be questioned since it was not drawn from a population’s register. We are nevertheless confident that, in the Swiss healthcare context, this recruitment method allowed for representative sampling. Indeed, few comparable characteristics of our patients with diabetes were close to those of a population-based cohort study conducted in the same area [[24]; P Marques-Vidal, personal communication]. Another limitation relating to the representativeness of the sample could be the moderate participation rate of patients (50%). This participation rate is however acceptable given that no reminders were sent. It was not feasible to ask pharmacies to collect additional information such as contact details of all eligible and possibly participating patients. Finally, analyses restricted to HbA1C-aware patients may have biased estimates of combined measures towards slightly better results (Table 4), without affecting item-by-item percentages. Indeed, HbA1C-aware patients were more likely than HbA1C unaware patients to report type 1 diabetes, present with a longer disease duration and insulin use, and were more likely to be younger, more educated and report higher income. However, HbA1C-aware and unaware patients with diabetes were not different in terms of gender, civil status, profession, insurance coverage, smoking, physical activity and self-reported health.

## Conclusions

In a wealthy country known for having good overall health indicators and long life expectancy, the results of this study showed that even if, on the whole, quality of diabetes care as measured by specific processes of care was acceptable, there remained room for further improvements. Indeed, special attention should be paid to diabetes specific risk screens, such as microalbuminuria check and eye assessment by ophtalmologist, participation in diabetes (self-management) education classes, dietary and physical activity recommendations, as well as multidisciplinary care, since they were all underused. These unmet targets should help set priorities for the further development and comprehensive monitoring of chronic disease management initiatives, such as those being implemented in several European and North American countries, as well as in Switzerland.

## Abbreviations

GDP: Gross domestic product; HbA1C: Hemoglobin A1C (glycated hemoglobin); OAD: Oral antidiabetic drugs; BMI: Body mass index.

## Competing interests

None of the authors have any conflict of interest to declare.

## Authors’ contributions

IPB conceived, designed and supervised the study; IPB also participated in the conduct of the study, performed statistical analysis and wrote the manuscript. JB helped in the design of the study, managed the study, helped in the statistical analysis and participated in drafting the manuscript. BB participated in the conception and design of the study, in the interpretation of the analyses and contributed to drafting the manuscript. All authors read and approved the final manuscript.

## Pre-publication history

The pre-publication history for this paper can be accessed here:

http://www.biomedcentral.com/1472-6963/13/232/prepub
